# The Sema3A receptor Plexin-A1 suppresses supernumerary axons through Rap1 GTPases

**DOI:** 10.1038/s41598-018-34092-5

**Published:** 2018-10-23

**Authors:** Nannan Wang, Pratibha Dhumale, Joanna Chiang, Andreas W. Püschel

**Affiliations:** 1 Institut für Molekulare Zellbiologie, Westfälische Wilhelms-Universität, Schloßplatz 5, D-48149 Münster, Germany; 2 Cells-in-Motion Cluster of Excellence, University of Münster, D-48149 Münster, Germany; 3 Present Address: Department of Cardiovascular and Renal Research, University of Southern Denmark, JB Winsløws Vej 21, 5000 Odense, Denmark

## Abstract

The highly conserved Rap1 GTPases perform essential functions during neuronal development. They are required for the polarity of neuronal progenitors and neurons as well as for neuronal migration in the embryonic brain. Neuronal polarization and axon formation depend on the precise temporal and spatial regulation of Rap1 activity by guanine nucleotide exchange factors (GEFs) and GTPases-activating proteins (GAPs). Several Rap1 GEFs have been identified that direct the formation of axons during cortical and hippocampal development *in vivo* and in cultured neurons. However little is known about the GAPs that limit the activity of Rap1 GTPases during neuronal development. Here we investigate the function of Sema3A and Plexin-A1 as a regulator of Rap1 GTPases during the polarization of hippocampal neurons. Sema3A was shown to suppress axon formation when neurons are cultured on a patterned substrate. Plexin-A1 functions as the signal-transducing subunit of receptors for Sema3A and displays GAP activity for Rap1 GTPases. We show that Sema3A and Plexin-A1 suppress the formation of supernumerary axons in cultured neurons, which depends on Rap1 GTPases.

## Introduction

Small GTPases of the Ras superfamily perform essential functions throughout neuronal development and in mature neurons^1^. The highly conserved Rap1 GTPases encoded by the *Rap1a* and *Rap1b* genes in mammals are required for the polarity of neuronal progenitors and neurons as well as for neuronal migration in the embryonic brain^1–5^. In the developing brain, newborn neurons that initially have a multipolar morphology become polarized by forming an axon and a leading process^1,6–9^. In culture, dissociated neurons from the embryonic hippocampus or cortex undergo a similar differentiation but polarize without the need for a patterned exogenous signal^7,10^. After attaching to the culture substrate neurons first extend several undifferentiated neurites (stage 2 of neuronal polarization) before one of them becomes an axon and extends rapidly. The inactivation of Rap1 GTPases impairs the formation of axons during cortical and hippocampal development *in vivo* and in cultured neurons^1,11^. Neuronal polarization and axon formation depend on the precise temporal and spatial regulation of Rap1 activity by GEFs and GAPs. Rapgef1 (also called C3G), Rapgef2 and Rapgef6 have been identified as the Rap1 GEFs that are required for the development of the neocortex and hippocampus^12–16^. However little is known about the GAPs that limit the activity of Rap1 GTPases during neuronal development^14^.

The plexins are integral membrane proteins with an intracellular domain that shows sequence similarity to GAPs with dual specificity for Ras and Rap GTPases^17–21^. The mutation of conserved arginine residues in this GAP domain is sufficient to abolish their activity *in vitro* and *in vivo*^20–23^. Plexins are receptors for the semaphorins, a large family of secreted and membrane-bound proteins that act as axon guidance signals but also perform important functions in other tissues^24–26^. The nine plexins in mammals can be subdivided into four subfamilies (PlexinA to -D). *In vitro* assays first showed that Plexin-B1 acts as a GAP for R- and M-Ras but not H-Ras^27–31^. Subsequently, a structural analysis of the GAP domain combined with biochemical assays demonstrated that Plexin-A1 and -C1 specifically regulate Rap1 and Rap2 GTPases^20,21,32–34^. The phenotype of plexin mutants confirmed that GAP activity is essential for their function *in vivo* and provided evidence for a regulation of Ras and Rap1 GTPases^22,23,35^.

The A-type plexins act as the signal-transducing subunit of receptors for the secreted Sema3A in a complex with Neuropilin-1 as the ligand binding subunit^24,36–40^. Semaphorins perform important functions not only during axon guidance but also in the regulation of neuronal migration and the formation of axons, dendrites and synapses^25,36,41–43^. Sema3A directs the orientation of axons and apical dendrites in the developing cortex^44–47^. It can also regulate the establishment of neuronal polarity by promoting the formation of dendrites and suppressing the extension of axons when neurons are cultured on a patterned substrate with stripes of immobilized Sema3A^48,49^. In cultures of sensory neurons from embryonic dorsal root ganglia, Sema3A accelerates the establishment of neuron polarity^50^. It remains to be investigated if plexins regulate the activity of Rap1 GTPases during neuronal polarization.

Here we show that Sema3A and Plexin-A1 suppress the formation of supernumerary axons in cultured neurons. Hippocampal but not cortical neurons from Sema3A knockout embryos form multiple axons in culture. While inactivation of Plexin-A1 results in the formation of supernumerary axons constitutively active Plexin-A1 inhibits axon formation. These effects can be rescued by the knockdown of Rap1B and the expression of active Rap1B, respectively, indicating that Plexin-A1 suppresses axon formation by regulating Rap1 GTPases.

## Results

### Hippocampal neurons from *Sema3a* knockout mice form multiple axons

**Figure 1:**
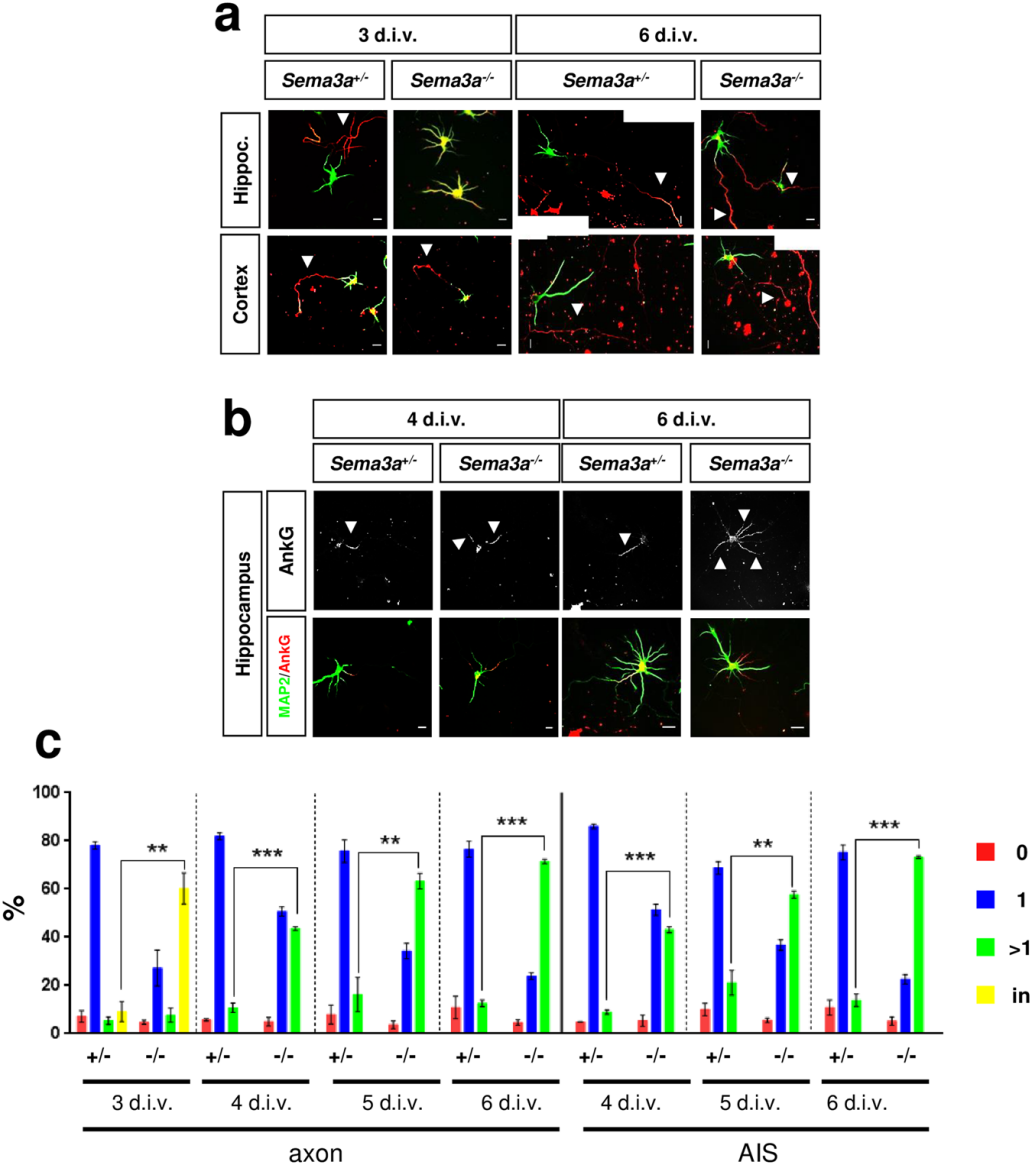
Hippocampal neurons from *Sema3a*^−/−^ embryos extend supernumerary axons in culture. (**a**–**c**) Cultures of hippocampal or cortical neurons from E17 *Sema3a*^+/−^ or *Sema3a*^−/−^ embryos were analyzed at 3, 4, 5 and 6 d.i.v. by staining with an anti-MAP2 (green, dendrites) and the Tau-1 (**a**, red, axons) or an anti-AnkG antibody (**b**, red, AIS marked by arrow heads). Representative images of hippocampal neurons at the indicated times in culture are shown. The scale bar is 25 μm. (**c**) The percentage of unpolarized hippocampal neurons without an axon or AIS (0, red), polarized neurons with a single axon or AIS (1, blue), neurons with multiple axons or AIS (>1, green) and neurons with neurites that are positive for both axonal and dendritic markers (in, yellow) is shown (Student’s t-test and two-way ANOVA; n = 3 independent experiments with >150 neurons per genotype; values are means ± s.e.m., **p < 0.01, ***p < 0.001 compared to control as indicated).

To determine if the establishment of neuronal polarity is affected in the absence of Sema3A neurons were isolated from the hippocampus and cortex of E17 *Sema3a*^−/−^ and *Sema3a*^+/−^ embryos and analyzed by staining with an anti-MAP2 and the Tau-1 antibody as markers for dendrites and axons, respectively (Fig. 1a,c). The majority (60 ± 5%) of the *Sema3*^−/−^ neurons extended several long neurites that were positive for both axonal and dendritic markers at day 3 days in culture (3 days *in vitro*, d.i.v.) compared to only 9 ± 4% in cultures from *Sema3a*^+/−^ embryos as control (Fig. 1c). To evaluate axon formation with a second axonal marker, cultures were analyzed at 4, 5 and 6 d.i.v. by staining with the Tau-1 or an anti-Ankyrin G (AnkG) antibody as a marker for the axonal initial segment (AIS) (Fig. 1b,c). A large proportion of the *Sema3a*^−/−^ neurons formed multiple Tau-1 positive axons (44 ± 1%) and multiple AIS (43 ± 1%) at 4 d.i.v. while only few were observed in *Sema3a*^+/−^ controls (axons: 11 ± 2%; AIS: 8 ± 1%). The number of *Sema3a*^−/−^ neurons with multiple Tau-1-positve axons increased to 63 ± 3% (AnkG: 58 ± 1%) at 5 d.i.v. and 71 ± 1% (AnkG: 73 ± 1%) at 6 d.i.v. (Fig. 1c). Thus, Sema3A-deficient neurons from the embryonic hippocampus showed defects in neuronal polarization. Neurons extended multiple neurites that initially display mixed axonal and dendritic characteristics at 3 d.i.v. but become supernumerary axons at 4 d.i.v. When cultures of cortical neurons from *Sema3a*^+/−^ and *Sema3a*^−/−^ embryos were analyzed no significant defect in neuronal polarization was observed at any of the analyzed time points (Fig. 1a,c). These results show that the knockout of *Sema3a* induces the formation of supernumerary axons by hippocampal but not cortical neurons.

### The knockdown of Sema3A induces cell-autonomous defects

**Figure 2:**
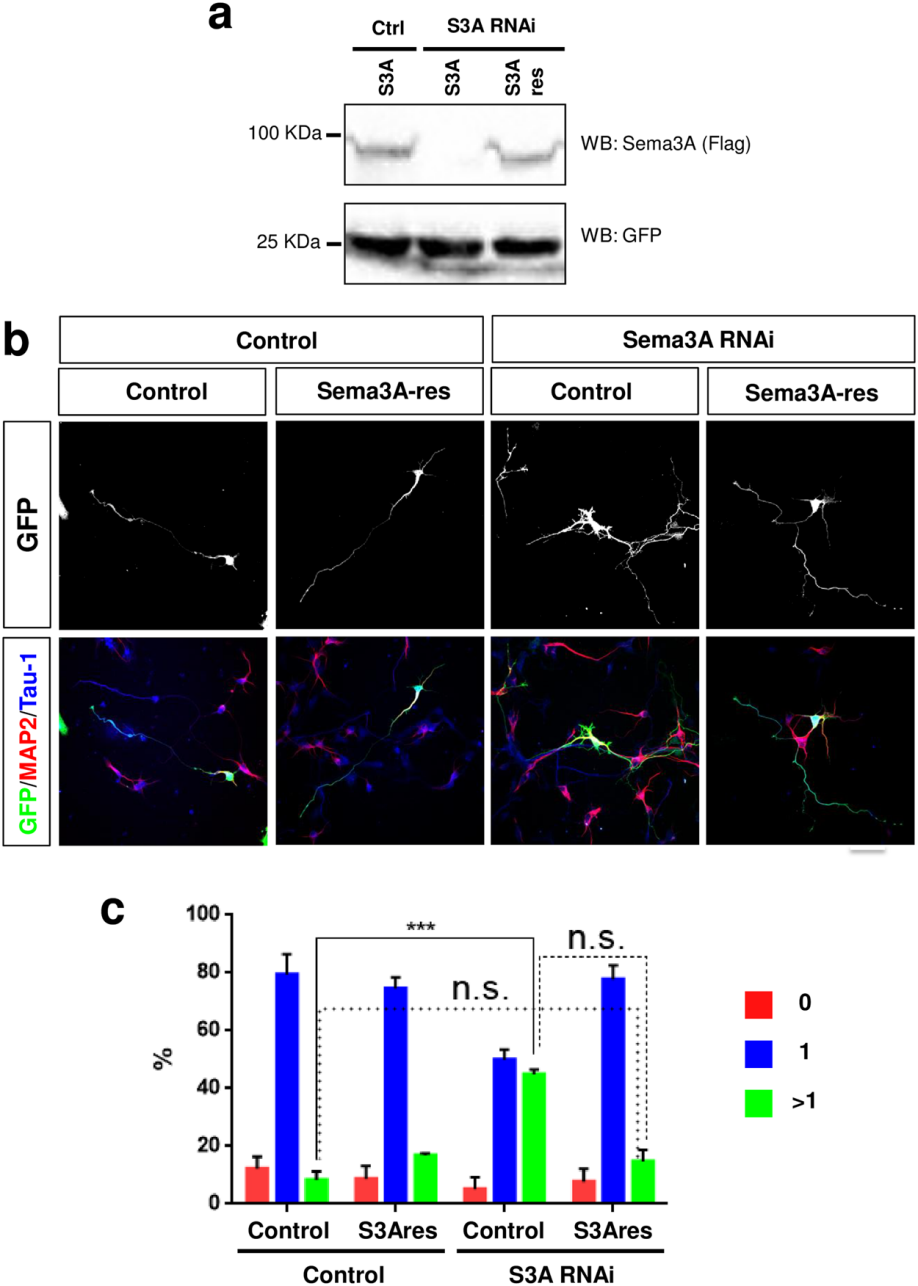
Knockdown of Sema3A induces the formation of supernumerary axons. (**a**) HEK 293 T cells were transfected with vectors for FLAG-Sema3A (S3A) or RNAi-resistant FLAG-Sema3A-res (S3Ares) and an shRNA directed against Sema3A (S3A RNAi) or pcDNA6.2-GW/EmGFP-miR (Ctrl). The expression of Sema3A and GFP was analyzed by Western blot (WB) using an anti-FLAG antibody. The molecular weight is indicated in kDa. (**b**) Hippocampal neurons from E18 rat embryos were transfected with vectors for GFP (green), an shRNA against Sema3A (Sema3A RNAi) or pcDNA6.2-GW/EmGFP-miR (control), and a vector for RNAi-resistant FLAG-Sema3A-res (Sema3A-res) or pBK-CMV (control) as indicated. Neurons were analyzed at 3 d.i.v. by staining with an anti-MAP2 (red, dendrites) and the Tau-1 antibody (blue, axon). Representative images of transfected neurons are shown. The scale bar is 25 μm. (**c**) The percentage of unpolarized neurons without an axon (0, red), polarized neurons with a single axon (1, blue) and neurons with multiple axons (>1, green) is shown (Student’s t-test and two-way ANOVA; n = 3, independent experiments with >150 neurons per experiment; values are means ± s.e.m, ***p < 0.001 compared to control as indicated; n.s., not significant).

Secreted Sema3A tightly binds to the cell surface of the producing cells and can act as an autocrine signal^51^. To investigate this possibility we used an miRNA expression vector to knock down Sema3A in cultured neurons. Since only a small number of neurons were transfected, a knockdown reduces the concentration of Sema3A in the medium only minimally compared to cultures from knockout embryos. The efficiency of the Sema3A knockdown construct was confirmed by Western blot after co-expression with tagged Sema3A in HEK 293 T cells (Fig. 2a) and by immunofluorescence staining of cultured hippocampal neurons (Suppl. Fig. S1a). Hippocampal neurons were transfected with the Sema3A knockdown vector and analyzed at 3 d.i.v. (Fig. 2b,c). Suppression of Sema3a induced the formation of multiple Tau-1 positive axons in 45 ± 1% of the neurons compared to 8 ± 2% in controls. By contrast, non-transfected neurons in the same culture where not affected by the knockdown indicating a cell-autonomous function of Sema3A in cultured neurons (Suppl. Fig. S1b). The phenotype of the Sema3A knockdown could be rescued by the expression of an RNAi-resistant Sema3A construct (Sema3A-res; Fig. 2) confirming the specificity of the knockdown. Only 15 ± 2% of the neurons that were co-transfected with the RNAi vector and the expression vector for Sema3A-res extended multiple axons and 78 ± 3% formed a single axon. These results suggest that Sema3A acts as an autocrine signal to suppress the formation of supernumerary axons.

### Plexin-A but not Plexin-B activity is required for neuronal polarity

**Figure 3:**
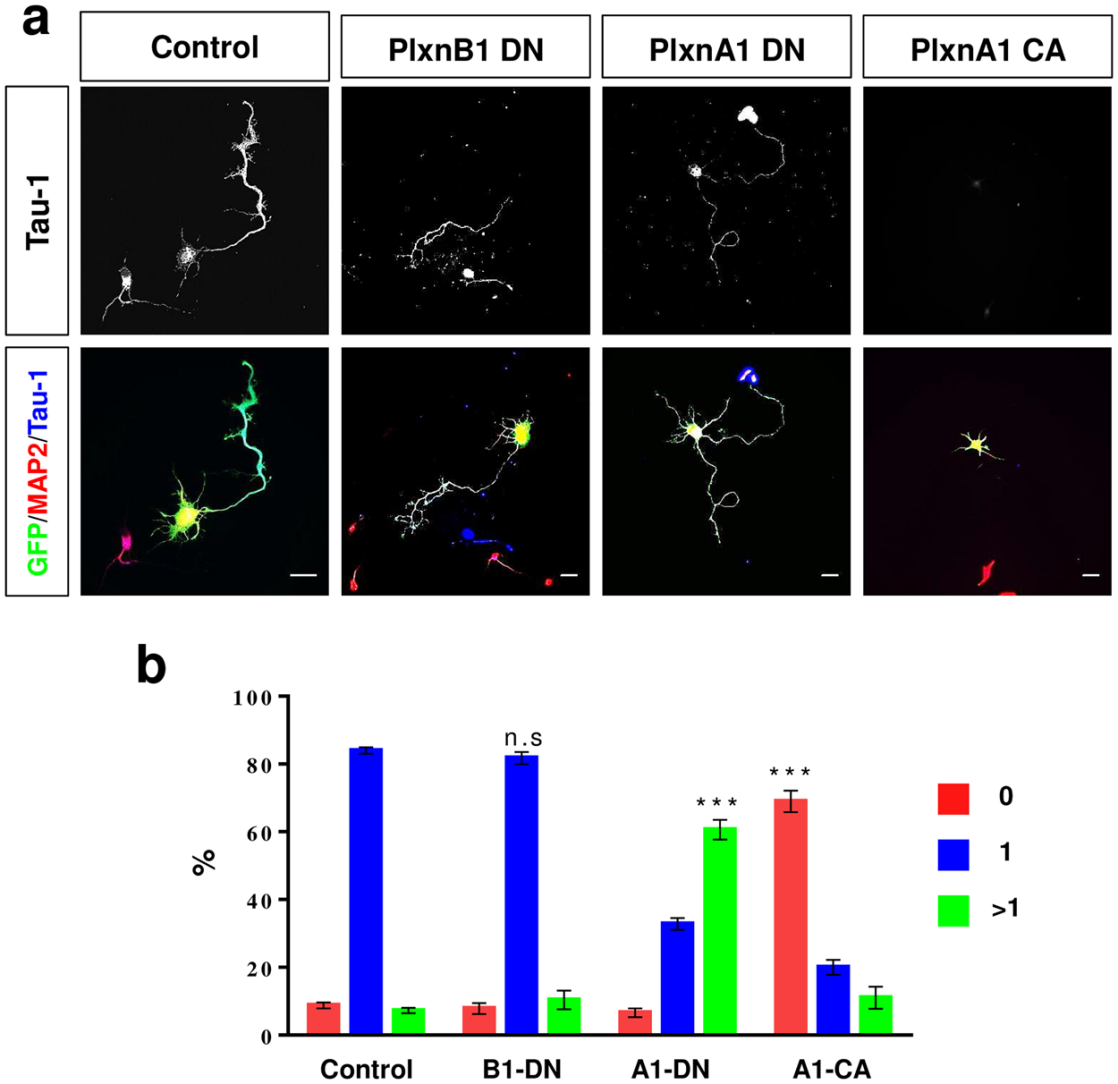
A-type plexins suppress the formation of axons. (**a**,**b**) Hippocampal neurons from E18 rat embryos were transfected with vectors for GFP (green, control), dominant-negative Plexin-B1Δcyt (PlxnB1-DN), Plexin-A1Δcyt (PlxnA1-DN) or constitutively active PlexinA1Δsema (PlxnA1-CA) and analyzed at 3 d.i.v. by staining with an anti-MAP2 (red, dendrites) and the Tau-1 antibody (blue, axon). Representative images of transfected neurons are shown. The scale bar is 25 μm. (**b**) The percentage of unpolarized neurons without an axon (0, red), polarized neurons with a single axon (1, blue) and neurons with multiple axons (>1, green) is shown (Student’s t-test and two-way ANOVA; n = 3, independent experiments with >150 neurons per experiment for; values are means ± s.e.m, ***p < 0.001 compared to control; n.s. not significant).

Sema3A acts through receptors that contain an A-type plexin as the signal transducing subunit^17,26,52,53^. To investigate whether plexins are involved in the regulation of neuronal polarization, we used a Plexin-A1 construct (Plexin-A1Δcyt) with a deletion of the intracellular domain that has a dominant-negative effect by forming non-functional receptor complexes^19,39,52^. Neurons from the hippocampus of E18 rat embryos were transfected with vectors for Plexin-A1Δcyt and analyzed at 3 d.i.v. by staining with an anti-MAP2 and the Tau-1 antibody (Fig. 3a). Expression of Plexin-A1Δcyt increased the number of neurons with multiple axons from 7 ± 1% in controls to 61 ± 2% (Fig. 3b). Deletion of the semaphorin domain releases Plexin-A1 from its auto-inhibited state and results in a constitutively active receptor (Plexin-A1Δsema)^54^. Expression of Plexin-A1Δsema had the opposite effect of dominant-negative Plexin-A1. The majority of neurons were negative for Tau-1 staining and the percentage of unpolarized neurons was increased to 69 ± 3% (control: 9 ± 1%; Fig. 3b). These results indicate that interfering with the function of A-type plexins disrupts the establishment of neuronal polarity.

To test a possible involvement of B-type plexins in axon formation, we used dominant-negative Plexin-B1Δcyt. After expression of Plexin-B1Δcyt, hippocampal neurons polarize normally and extend a single Tau-1 positive axon (Fig. 3a). No significant difference was detectable between controls (neurons with a single axon: 85 ± 4%) and the expression of Plexin-B1Δcyt (82 ± 25%; Fig. 3b). Thus, inhibition of Plexin-B1 has no effect on axon formation.

### Suppression of Plexin-A1 induces multiple axons

**Figure 4:**
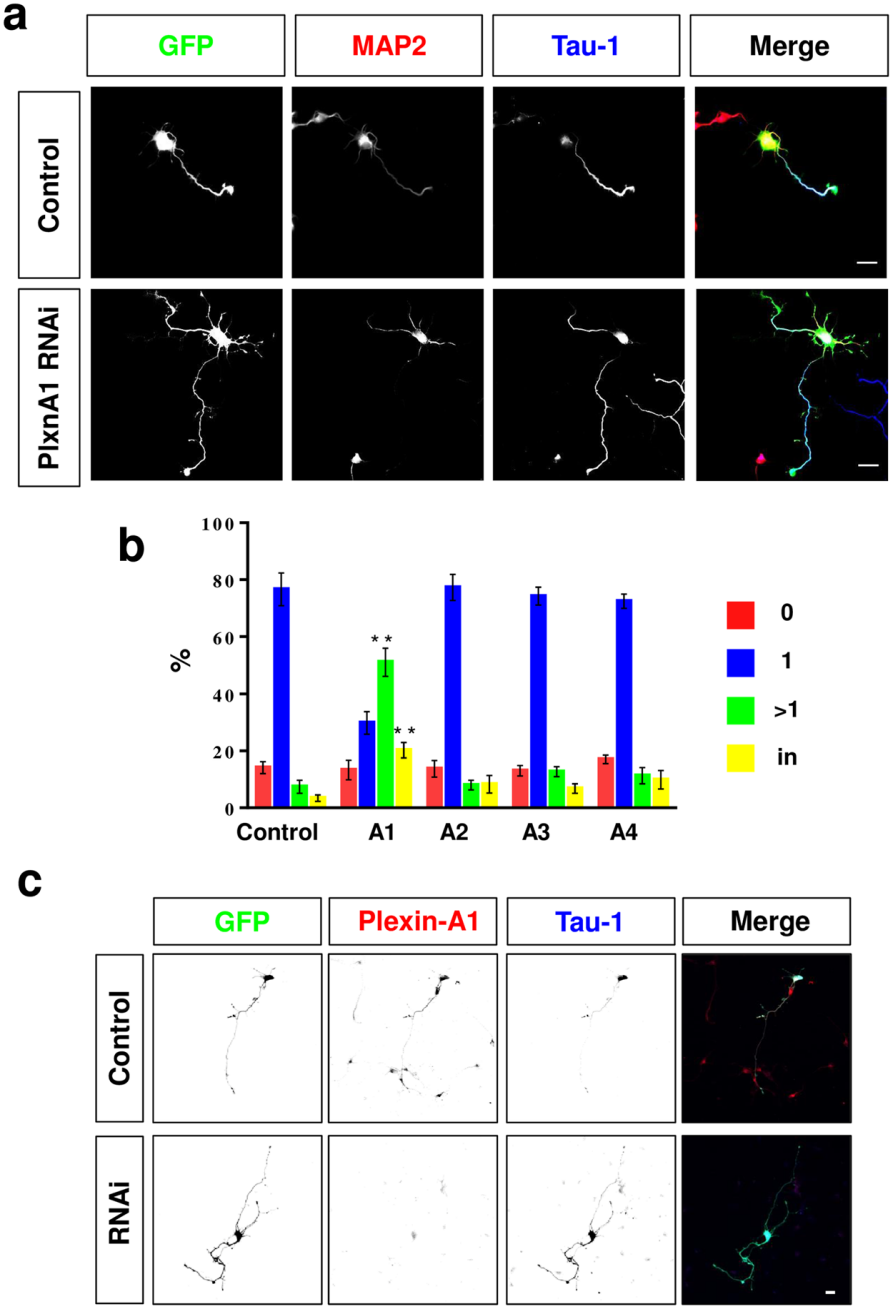
Knockdown of Plexin-A1 induces the formation of supernumerary axons. (**a**,**b**) Hippocampal neurons from E18 rat embryos were transfected with vectors for GFP (green) and shRNAs against Plexin-A1, -A2. -A3, or -A4 or pSUPER (control) and analyzed at 3 d.i.v. by staining with an anti-MAP2 (red, dendrites) and the Tau-1 antibody (blue, axon). Representative images of transfected neurons are shown. The scale bar is 25 μm. (**b**) The percentage of unpolarized neurons without an axon (0, red), polarized neurons with a single axon (1, blue), neurons with multiple axons (>1, green) and neurons with neurites that are positive for both axonal and dendritic markers (in, yellow) is shown (Student’s t-test and two-way ANOVA; n = 3, independent experiments with >150 neurons per experiment; values are means ± s.e.m, **p < 0.01 compared to control). (**c**) Hippocampal neurons from E18 rat embryos were transfected with vectors for GFP (green) and an shRNA against Plexin-A1 (RNAi) or pSUPER (control) and analyzed at 3 d.i.v. by staining with an anti-Plexin-A1 (red) and the Tau-1 antibody (blue, axon). Representative images of transfected neurons are shown. The scale bar is 25 μm.

The dominant-negative Plexin-A1Δcyt construct may affect all receptors that include a member of the Plexin-A subfamily. To identify which A-type plexin regulates neuronal polarity, we used established RNAi vectors for PlexinA1 - A4^41^. Rat hippocampal neurons were transfected with these RNAi vectors and axon formation was analyzed at 3 d.i.v. (Fig. 4a,b). After knockdown of Plexin-A1, the number of neurons with multiple axons increased from 7 ± 2% in controls to 51 ± 4% (Fig. 4b). In addition, 20 ± 4% of the neurons formed neurites that were positive for both axonal and dendritic markers. By contrast, after knockdown of Plexin-A2, -A3 or -A4 no significant increase in the extension of supernumerary axons was observed (neurons with a single axon: Plexin-A2: 77 ± 4%, Plexin-A3: 74 ± 3%; Plexin-A4: 72 ± 2%; control: 77 ± 5%). Staining with an anti-Plexin-A1 antibody confirmed that the knockdown efficiently suppressed the expression of Plexin-A1 that is present in all neurites of polarized hippocampal neurons (Fig. 4c). The formation of supernumerary axons after knockdown of Plexin-A1 could be reversed by co-expressing murine Plexin-A1 that contains mismatches in the RNAi target site compared to the rat sequence confirming the specificity of the Plexin-A1 knockdown in rat neurons (Suppl. Fig. S2). These results show that Plexin-A1 is required during neuronal polarization to prevent the formation of supernumerary axons.

### Plexin-A1 acts upstream of Rap1 GTPases during neuronal polarization

**Figure 5:**
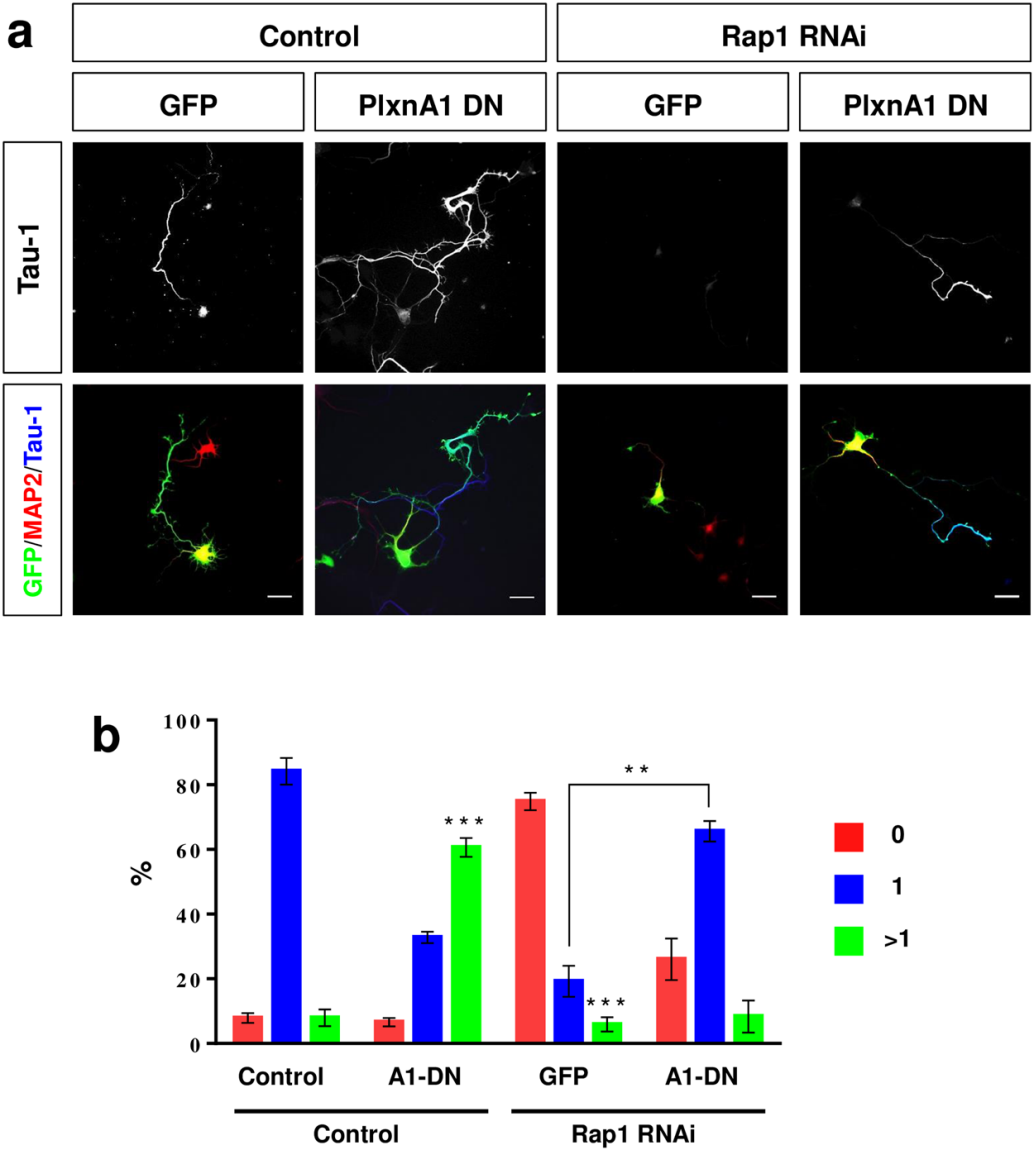
Knockdown of Rap1B counteracts the induction of supernumerary axons by dominant-negative Plexin-A1. (**a**) Hippocampal neurons from E18 rat embryos were transfected with vectors for GFP (green, control), an shRNA against Rap1B or pSHAG-1 (control) and Plexin-A1Δcyt (PlxnA1-DN) or pBK-CMV (control). The establishment of neuronal polarity was analyzed at 3 d.i.v. by staining with an anti-MAP2 (red, dendrites) and the Tau-1 antibody (blue, axons). Representative images of transfected neurons are shown. The scale bar is 25 μm. (**b**) The percentage of unpolarized neurons without an axon (0, red), polarized neurons with a single axon (1, blue) and neurons with multiple axons (>1, green) is shown (Student’s t-test and two-way ANOVA; n = 3, independent experiments with >150 neurons per experiment for; values are means ± s.e.m, **p < 0.01; ***p < 0.001 compared to control and as indicated.

Structural and biochemical analyses showed that Plexin-A1 regulates Rap1 GTPases that are required for the formation of axons^1,11,20,21^. The inactivation of the Rap1 GAP Plexin-A1 may, therefore, result in the extension of supernumerary axons due to increased Rap1 activity. To investigate this possibility, we tested if the formation of multiple axons after expression of dominant-negative Plexin-A1Δcyt can be blocked by a knockdown of Rap1B. Hippocampal neurons were co-transfected with vectors for Plexin-A1Δcyt and an shRNA directed against Rap1B^11^ and analyzed at 3 d.i.v. (Fig. 5a). After knockdown of Rap1B, only 19 ± 4% of the transfected neurons extended an axon compared to 84 ± 3% in controls (Fig. 5b) as shown before^11^. The induction of supernumerary axons by Plexin-A1Δcyt was prevented by the knockdown of Rap1B (unpolarized neurons: 26 ± 5%, neurons with a single axon: 66 ± 3%, neurons with multiple axons: 8 ± 4%) indicating that it depends on Rap1B. The specificity of the Rap1B knockdown was verified by co-expression of an Rap1B construct with mismatches in the RNAi target site that rescued the loss of axons after Rap1B knockdown (Suppl. Fig. S3).

**Figure 6:**
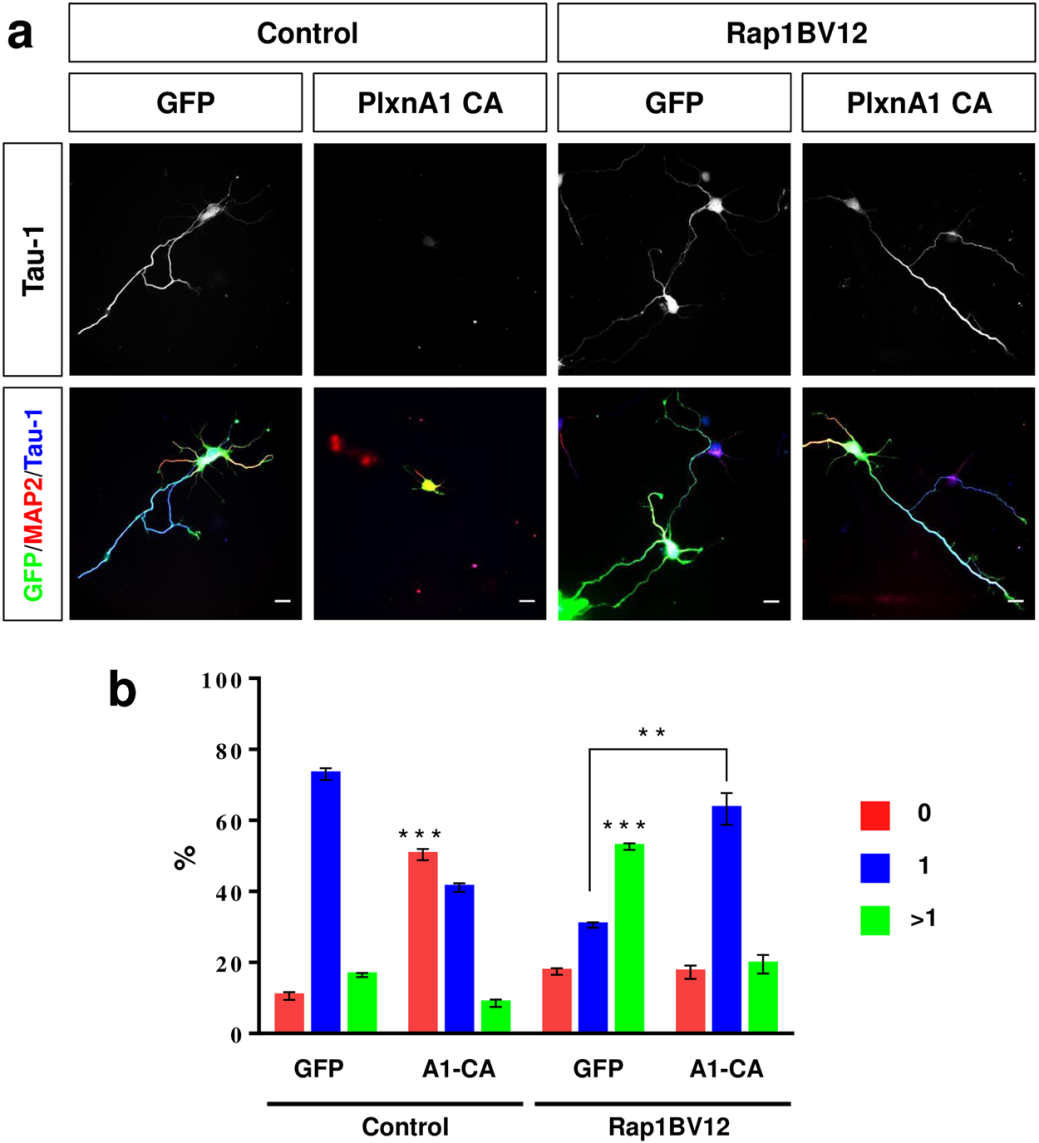
Active Rap1B recues the suppression of axon formation by constitutively active Plexin-A1. (**a**) Hippocampal neurons from E18 rat embryos were transfected with vectors for GFP (green) and Plexin-A1Δsema (PlxnA1-CA), Rap1BV12 or a combination of both and analyzed at 3 d.i.v. by staining with an anti-MAP2 (red, dendrites) and the Tau-1 antibody (blue, axon). pBK-CMV and pSHAG-1 were transfected as control. Representative images of transfected neurons are shown. The scale bar is 25 μm. (**b**) The percentage of unpolarized neurons without an axon (0, red), polarized neurons with a single axon (1, blue) and neurons with multiple axons (>1, green) is shown (Student’s t-test and two-way ANOVA; n = 3, independent experiments with >150 neurons per experiment; values are means ± s.e.m, **p < 0.01; ***p < 0.001 compared to control and as indicated).

The expression of constitutively active Plexin-A1Δsema has an effect that is similar to that of a Rap1B knockdown and leads to the loss of axons. We tested if this phenotype can be rescued by the expression of active Rap1BV12. As reported before^11^, expression of Rap1BV12 increased the number of hippocampal neurons with multiple axons (53 ± 1%) compared to controls (17 ± 1%) while expression of Plexin-A1Δsema increased the number of unpolarized neurons (51 ± 2%, control: 11 ± 1%; Fig. 6a,b). The suppression of axon formation by PlexinA1Δsema could be rescued by co-expression of Rap1BV12, which increased the percentage of polarized neurons with a single axon from 43 ± 1% (only PlexinA1Δsema) to 63 ± 4% (PlexinA1Δsema and Rap1BV12, Fig. 6b). These results indicate that Plexin-A1 acts upstream of Rap1 GTPases and restricts their activity.

**Figure 7:**
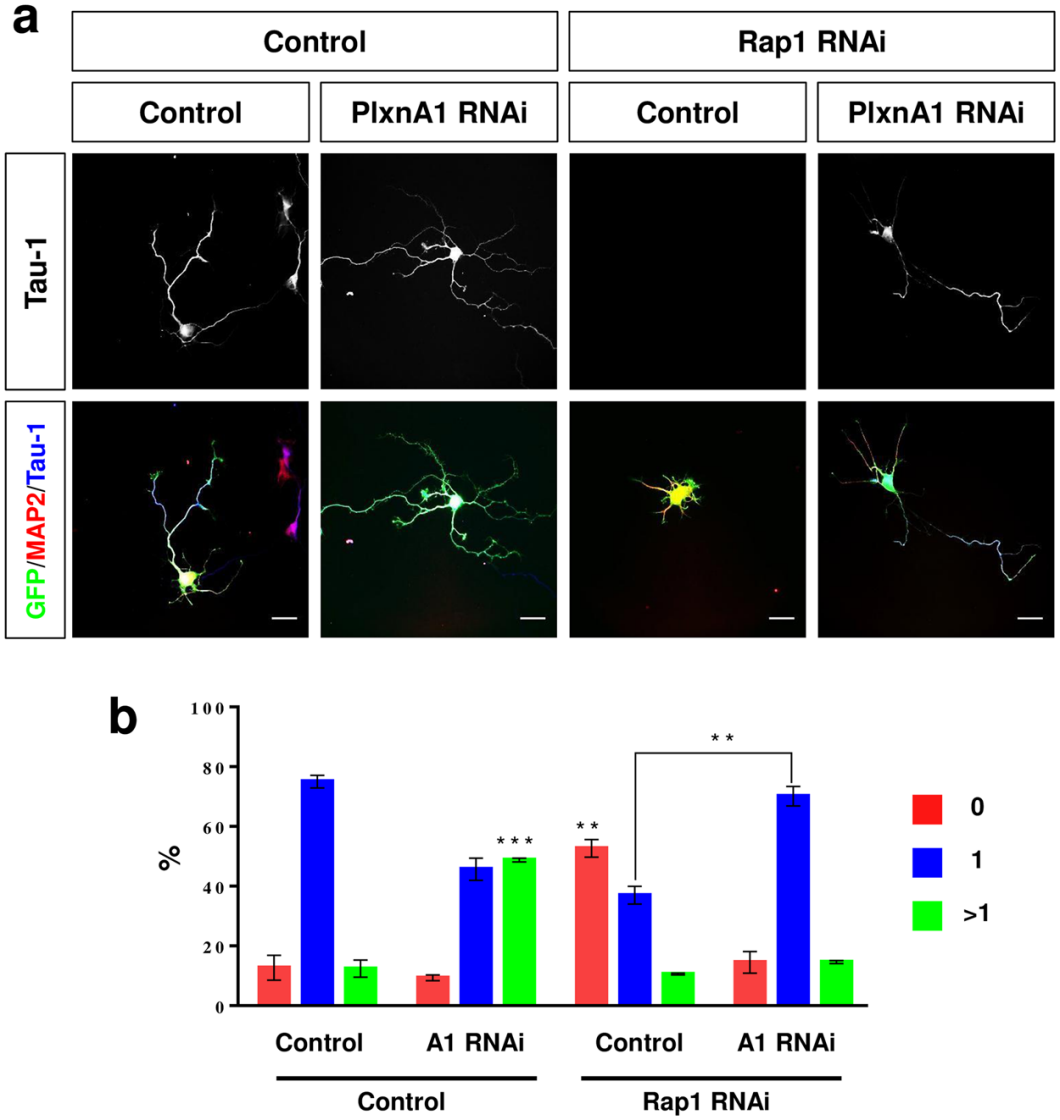
Rap1B is required for the induction of supernumerary axons by the knockdown of Plexin-A1 in hippocampal neurons. (**a**) Hippocampal neurons from E18 rat embryos were transfected with vectors for GFP (green) and Plexin-A1Δsema (PlxnA1-CA), Rap1BV12, pSHAG-1 (control) or a combination of both and analyzed at 3 d.i.v. by staining with an anti-MAP2 (red, dendrites) and the Tau-1 antibody (blue, axon). Representative images of transfected neurons are shown. The scale bar is 25 μm. (**b**) The percentage of unpolarized neurons without an axon (0, red), polarized neurons with a single axon (1, blue) and neurons with multiple axons (>1, green) is shown (Student’s t-test and two-way ANOVA; n = 3, independent experiments with >150 neurons per experiment for; values are means ± s.e.m, **p < 0.01; ***p < 0.001 compared to control and as indicated).

To confirm that Rap1 GTPases act downstream of Plexin-A1 we tested if the formation of supernumerary axons caused by the knockdown of Plexin-A1 can be prevented by the knockdown of Rap1B. After transfection of hippocampal neurons with the shRNA vector directed against Plexin-A1, 48 ± 1% of the transfected neurons extended multiple axons (Fig. 7a,b). This number was reduced to 14 ± 1% when the shRNA against Rap1B was cotransfected with the Plexin-A1 shRNA and the majority of the neurons (70 ± 3%) extended a single Tau-1 positive axon. These results show that Rap1B is required for the induction of supernumerary axons by the knockdown of Plexin-A1. Taken together, our results show that Plexin-A1 acts upstream of Rap1B and is required to restrict its activity in hippocampal neurons.

### Rap1B acts downstream of Sema3A in hippocampal neurons

**Figure 8:**
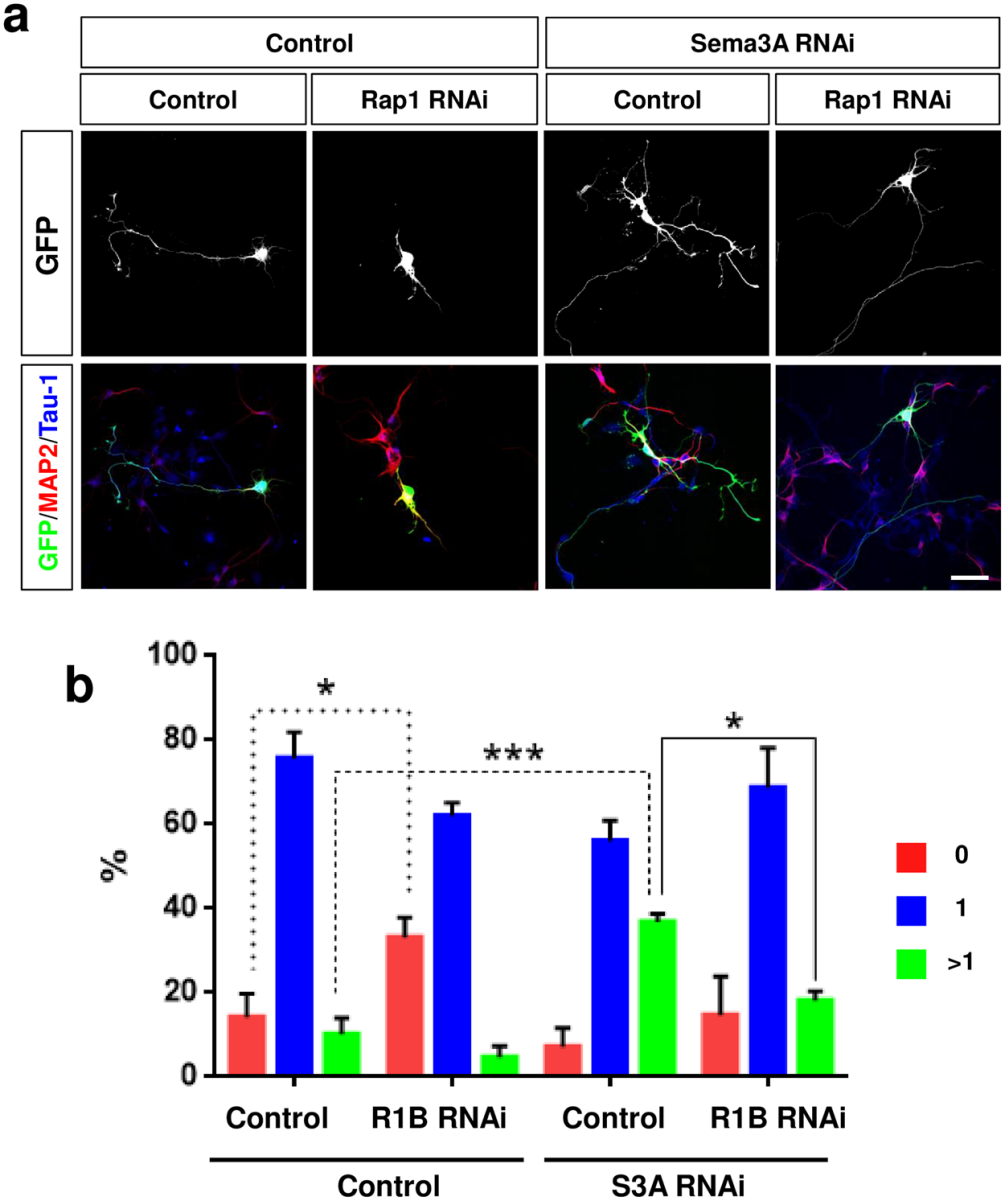
Rap1B acts downstream of Sema3A in hippocampal neurons. (**a**) Hippocampal neurons from E18 rat embryos were transfected with vectors for GFP (green), an shRNA against Sema3A (S3A RNAi) or pcDNA6.2-GW/EmGFP-miR (control) and an shRNA against Rap1B (R1B RNAi) or pSHAG-1 (control) as indicated. Neurons were analyzed at 3 d.i.v. by staining with an anti-MAP2 (red, dendrites) and the Tau-1 antibody (blue, axon). Representative images of transfected neurons are shown. The scale bar is 25 μm. (**b**) The percentage of unpolarized neurons without an axon (0, red), polarized neurons with a single axon (1, blue) and neurons with multiple axons (>1, green) is shown (Student’s t-test and two-way ANOVA; n = 3, independent experiments with >150 neurons per experiment for; values are means ± s.e.m, ***p < 0.001 compared to control as indicated).

Sema3A-deficient neurons extend multiple axons similar to the phenotype of neurons transfected with a vector for constitutively active Rap1BV12. To investigate if Rap1B acts downstream of Sema3A we tested if the knockdown of Rap1B prevents the formation of supernumerary axons after knockdown of Sema3A. 32 ± 5% of the transfected neurons extended multiple axons (Fig. 8) that were also positive for Rap1 after the knockdown of Sema3A (Suppl. Fig. S4). This number was reduced to 18 ± 2% with the majority of the neurons (67 ± 6%) extending a single Tau-1 positive axon when Rap1B was knocked down together with Sema3A. These results show that the formation of supernumerary axons in Sema3A-deficient neurons depends on Rap1B.

## Discussion

Our results suggest that Sema3A acts as an autocrine signal through Plexin-A1, which restricts the activity of Rap1 and thereby prevents the formation of supernumerary axons. Hippocampal neurons from *Sema3a*^−/−^ knockout mice form multiple axons in culture. Suppressing Plexin-A1 function by a dominant-negative construct or a knockdown had a similar effect while expression of constitutively active Plexin-A1 blocked axon formation. The defects in neuronal polarity were rescued by the knockdown of Rap1B and the expression of active Rap1BV12, respectively, which is consistent with the activity of Plexin-A1 as a GAP for Rap1^20,21^. These results indicate that Rap1 GTPases act downstream of Plexin-A1 and are required for its function to suppress the formation of supernumerary axons.

Our results are consistent with previous reports that Sema3A suppresses axon formation^48,49^. Hippocampal neurons express Sema3A^44,55^ that remains tightly bound to the surface of producing cells after secretion by binding to proteoglycans^51^. A knockdown of Sema3A induces the formation of supernumerary axons only in transfected neurons while non-transfected neurons in the same culture are not affected. The co-expression of an RNAi-resistant Sema3A rescues the Sema3A knockdown and prevents the formation of supernumerary axons. These results suggest that Sema3A acts primarily in a cell-autonomous manner as an autocrine factor as described for other cell types^56–60^. *Sema3a* knockout neurons extend multiple long neurites that are initially positive for both axonal and dendritic markers at 3 d.i.v. before they become axons by 4 d.i.v. while most neurons extend multiple axons already at 3 d.i.v. after expression of dominant-negative Plexin-A1 and knockdown of Plexin-A1 or Sema3A. The major reason for this difference in the time course probably is the time point at which Sema3A/Plexin-A1 signaling is inhibited. Sema3A acts like a global inhibitory signal that suppresses axon formation^48,61^. It is absent from the beginning in knockout neurons while it is transiently produced after a knockdown, which may explain why knockout neurons initially extend neurites with both dendritic and axonal properties before these become axons.

In contrast to Plexin-A1, we did not observe defects in neuronal polarity after expression of dominant-negative Plexin-B1. The analysis of Plexin-B1 suggested that it acts as a GAP for R-Ras and M-Ras^27–30^. Different Ras GTPases have been implicated in axon formation^29,62,63^. The activation of Plexin-B1 by Sema4D down-regulates the activity of R- and M-Ras in the growth cones of axons and dendrites of cultured hippocampal neurons, respectively^27,30–32^. However, neither single nor double knockouts of Ras GTPases show defects in brain development^64^, indicating that they are not required for neuronal polarity *in vivo* or that their loss is compensated by other Ras GTPases. *In vivo* studies indicate that B-type plexins perform different functions during neuronal development^22,65–69^. Knockout of *Plxnb1* and *Plxnb2* results in a reduced proliferation of neural progenitors and cortical thinning, which could indicate a function in regulating the orientation of the mitotic spindle as shown for epithelial cells^22,67,69^. Further studies are required to elucidate which GTPases are regulated by the different plexins *in vivo* and what determines their specificity. It also remains to be investigated whether other GAPs are involved in mediating the function of Sema3A or the regulation of Rap1 GTPases during neuronal polarization.

Unlike in hippocampal neurons, axon formation was not affected in cortical neurons. The differential effects of the *Sema3a* knockout may result from differences in the expression of semaphorin receptors and other semaphorins like Sema3C may act in the developing neocortex. Sema3C is expressed in migrating neurons in the cortex and its overexpression interferes with their polarization and radial migration^43^. Sema3A could be required specifically in hippocampal neurons to suppress axon formation. A difference between hippocampus and cortex was observed also for the *Rap1a*;*Rap1b* knockout^1^. The conditional knockout of Rap1 GTPases at different time points showed that an inactivation late during neuronal polarization interferes with axon formation in the hippocampus but not in the cortex. The hippocampus-specific functions of Sema3A and Rap1 GTPases could be linked to the extended time that hippocampal neurons remain in the multipolar phase of migration, which may require additional mechanisms to delay axon formation^70^.

## Materials and Methods

### Sema3a knockout mice

*Sema3a*^+/−^ mice^71^ were maintained in a C57Bl/6 background. Genotyping was performed using the primers 5′-ATGGTTCTGA TAGGTGAGGC ATGG-3′, 5′-GTTCTGCTCC CGGCTCTAAA TCTC-3′ and 5′-AGGCAAACTA TGCAAACGG AAAG-3′^72^. Mice were housed at four to five per cage with a 12 h light/dark cycle (lights on from 07:00 to 19:00 h) at constant temperature (23 °C) with *ad libitum* access to food and water. All animal protocols were carried out in accordance with the relevant guidelines and regulations and approved by the Landesamt für Natur, Umwelt und Verbraucherschutz Nordrhein-Westfalen.

### Cell culture and transfection

Cultures of hippocampal and cortical neurons were prepared from E17 *Sema3a* mouse embryos as described before^1^. Hippocampal neurons from E18 rat embryos were prepared and transfected by calcium phosphate co-precipitation as described previously^1^. Dissociated neurons were plated at a density as 65,000 cells per well of a 24-well plate containing cover slips coated with poly-L-ornithine (15 μg/ml, SigmaAldrich). Neurons were cultured at 37 °C and 5% CO_2_ for 3–6 days in Neurobasal medium (Invitrogen) with supplements. An excess of the expression and knockdown vectors were combined with a small amount of pEGFP-N3 (Clontech) to label transfected neurons.

### Immunofluorescence staining

Neurons were fixed with 4% paraformaldehyde/15% sucrose in phosphate buffered saline (PBS) for 15 min at RT and permeabilized with 0.1% Triton X-100/0.1% Na-Citrate/PBS for 10 min on ice. Cells were incubated in 10% normal goat serum (PAN Biotech) in PBS for 1 h at RT, stained with primary antibody overnight at 4 °C and secondary antibody for 90 min, and mounted using Mowiol (SigmaAldrich). Neuronal morphology and axon formation were analyzed as described before using a Zeiss Axiophot microscope equipped with a Visitron CCD camera and the SPOT Advanced Imaging software or a Zeiss LSM 700 using the ZEN (black edition) software^1^. Image analysis was done using ImageJ 1.45 s (NIH), ZEN (black edition) and Adobe Photoshop CS5. All statistical data are means ± s.e.m. from at least three times independent experiments. Statistical significance was determined using the Student’s t-test.

### Antibodies

The following antibodies were used: mouse Tau-1 (Chemicon, MAB3420, 1:200), mouse anti-MAP2 (SigmaAldrich, M4403, 1:1500), rabbit anti-MAP2 (Abcam, ab32454, 1:1000), mouse anti-Ankyrin-G (Antibodies Inc., 75–146, 1:100), rabbit anti-Plexin-A1 (Abcam, ab23391, 1:1000), mouse anti-GFP (Covance, MMS-118P, 1:1000), mouse anti-FLAG M2 (SigmaAldrich, F3165, 1:1000), anti-Rap1 (Upstate, #07–916, 1:200), anti-Sema3A (Abcam, ab23393, 1:200), SMI-312 (BioLegend, 837904, 1:200) and goat secondary antibodies labeled with Alexa-350 (Molecular Probes, 1:200), −488 (1:800) or −594 (1:800). Nuclei were stained with Hoechst 33342 (Molecular Probes, 1:6000).

### Plasmids

Published shRNA vectors were used for a knockdown of different A-type plexins (target sequences: Plexin-A1: 5′-CCGTATTTAC AAGCTGTCG-3′; Plexin-A2: 5′-GCGCAAGTCT AGGGAAAAT-3′; Plexin-A3: 5′-GTGCGGGTTC GGCCTAATA-3′; Plexin-A4: 5′-AGATGCTGCT TATAGAC TA-3′)^41^. The Plexin-A1 shRNA is specific for the rat sequence^41^. For rescue experiments, an expression vector for murine Plexin-A1^19^ that contains mismatches in the shRNA target site compared to the rat sequence was used. The shRNA vector targeting Rap1B and the expression vectors for Rap1BV12 and Flag-Sema3A have been described before^11,73^. A vector for RNAi-resistant Rap1B (Rap1B-res) was constructed by site-directed mutagenesis of the shRNA target site^11^ to introduce mismatches using the QuikChange Site-Directed Mutagenesis kit (Stratagene) with the oligonucleotides 5′-GTTGTAGGAA AAGAACAGGG TCAAAACCTA GCAAGACAG-3′ and 5′-CTGTCT TGCT AGGTTTTGAC CCTGTTCTTT TCCTACAAC-3′. The miRNA vector targeting Sema3A with the target sequence 5′-TTCCGGGAAC CAACAACTATT-3′ was generated using the BLOCK-iT Pol II miRNA Expression Vector Kit (Invitrogen), inserted into pcDNA6.2-GW/EmGFP-miR and confirmed by sequencing. A vector for RNAi-resistant FLAG-Sema3A (Sema3A-res) was constructed by site-directed mutagenesis using the QuikChange Site-Directed Mutagenesis kit (Stratagene) with the oligonucleotides 5′-GAAATGACCG TCTTCCGTGA ACCGACAACC ATTTCAGCAATG-3′ and 5′-CATTGCTGAA ATGGTTGTCG GTTCACGGAA GACGGTCATT TC-3′. pEGFP-N3 (Clontech) was used as transfection control. The dominant-negative Plexin-A1 construct pBK-CMV-VSV-Plexin-A1Δcyt with a deletion of the intracellular domain and the constitutively active pBK-CMV-VSV-Plexin-A1Δsema with a deletion of the semaphorin domain were constructed as described before^19,39,52,54^.

### Transfection of HEK 293T cells and Western blot

HEK 293 T cells were transfected using the calcium phosphate co-precipitation method as described previously^74^. Transfected HEK 293 T cells were lysed in Tris/HCl 50 mM, pH 7,4, NaCl 150 mM, DTT 1 mM, MgCl_2_ 1,5 mM, EDTA 4 mM, glycerol 10% (v/v), Triton X-100 1% (v/v), cOmplete protease inhibitor (Sigma-Aldrich) and expression of Sema3A analyzed by Western blot using horseradish peroxidase conjugated secondary antibodies (Dianova, 1:3000). Peroxidase activity was visualized by the enhanced chemiluminescence detection system (Uptima, Interchim UP99619A) using the ChemiDoc^TM^ MP imaging system (Bio-Rad).

### Quantification

The establishment of neuronal polarity was quantified by counting the number of transfected (GFP-positive) neurons that did not extend an axon (unpolarized neuron), formed a single axon positive for Tau-1 (polarized neuron), multiple axons positive for Tau-1 (multiple axons) or multiple axons positive for Tau-1 and MAP2 (indeterminate phenotype). Statistical analyses were done using the GraphPad Prism 6.0 software. Statistical significance was calculated for at least three independent experiments using two-way ANOVA with Tukey’s multiple comparison test and Student’s t-Test for parametric data sets. Significance was defined as: p > 0.05, n.s.; *p < 0.05, **p < 0.01, ***p < 0.001, ****p < 0.0001.

## Electronic supplementary material

Supplementary Figures S1 - S5
