# Supplementary material for: The Sema3A receptor Plexin-A1 suppresses supernumerary axons through Rap1 GTPases
Source: Sci Rep. 2018 Oct 23;8:15647. doi: 10.1038/s41598-018-34092-5 (PMC6199275; doi:10.1038/s41598-018-34092-5)
Supplement: Supplementary file 1 — Supplementary Figures S1 - S5 [file 41598_2018_34092_MOESM1_ESM.pdf]

The Sema3A receptor Plexin-A1 suppresses supernumerary axons through Rap1 GTPases

Supplementary material

Nannan Wang[1], Pratibha Dhumale[1,2,3,*], Joanna Chiang[1,*] Andreas W. Püschel[1,2]

[1]Institut für Molekulare Zellbiologie, Westfälische Wilhelms-Universität, Schloßplatz 5, D-48149 Münster, Germany

[2]Cells-in-Motion Cluster of Excellence, University of Münster, D-48149 Münster, Germany

[3]Current address: Department of Cardiovascular and Renal Research, University of Southern Denmark, JB Winsløws Vej 21, 5000 Odense, Denmark

[*] These authors contributed equally to this work

Supplementary Figures

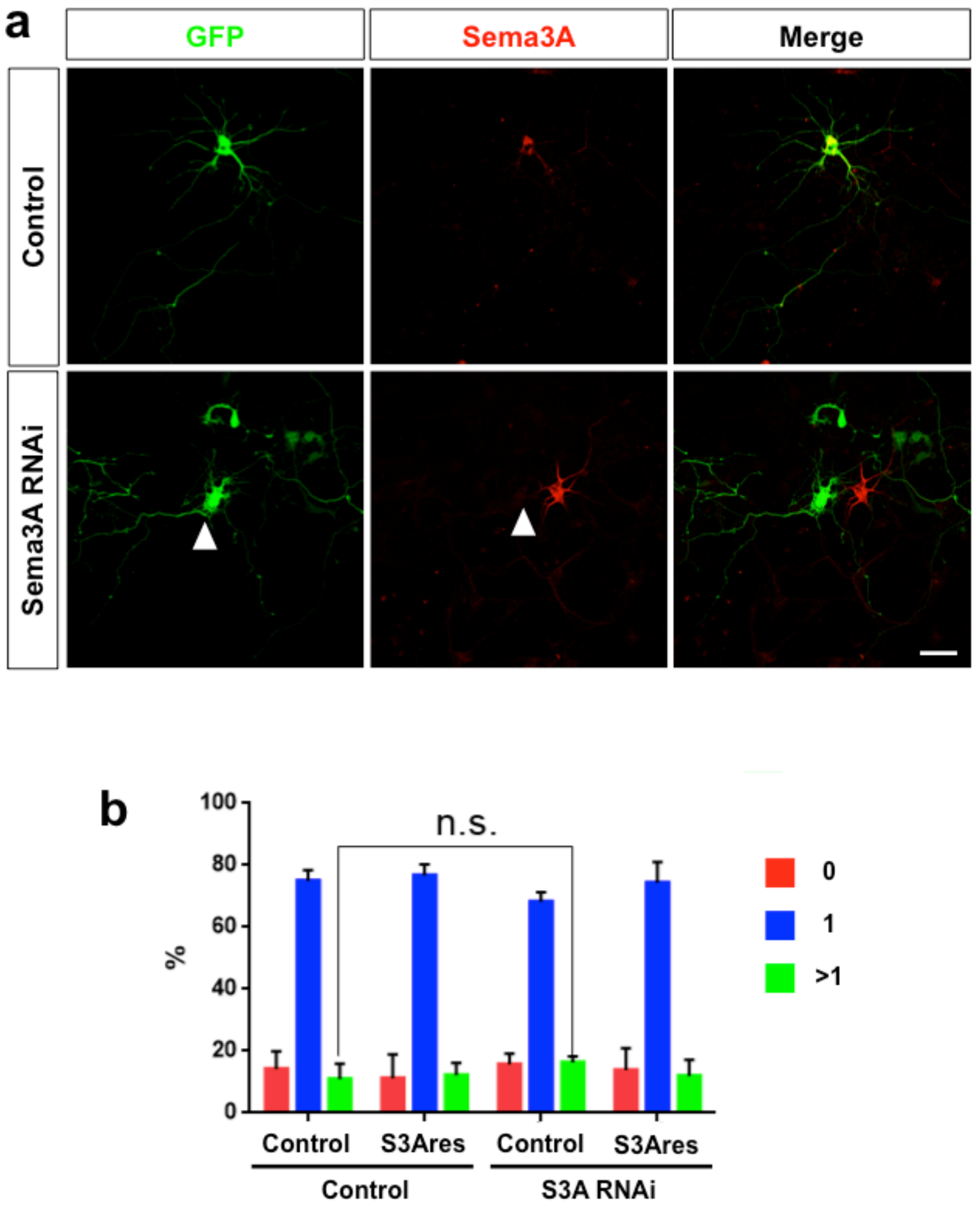

Figure S1. Knockdown of Sema3A induces the formation of supernumerary axons. (**a**) Hippocampal neurons from E18 rat embryos were transfected with vectors for GFP (green) and an shRNA against Sema3A (Sema3A RNAi) or pcDNA6.2-GW/EmGFP-miR (control) and stained at 3 d.i.v. with an anti-Sema3A antibody (red). The arrowhead marks a transfected neuron that is negative for Sema3A while the neighboring non-transfected neuron is positive for Sema3A. (**b**) Hippocampal neurons from E18 rat embryos were transfected with vectors for GFP and an shRNA against Sema3A (S3A RNAi) or pcDNA6.2-GW/EmGFP-miR (control) and a vector for RNAi-resistant FLAG-Sema3A-res (S3A-res) or pBK-CMV (control) as indicated (see Fig. 2). Non-transfected neurons from the same cultures as shown in Fig. 2 were analyzed at 3 d.i.v.. The percentage of non-transfected, unpolarized neurons without an axon (0, red), polarized neurons with a single axon (1, blue) and neurons with multiple axons (>1, green) is shown (Student's t-test and two-way ANOVA; n=3, independent experiments with>150 neurons per experiment for; values are means ± s.e.m.; n.s., not significant).

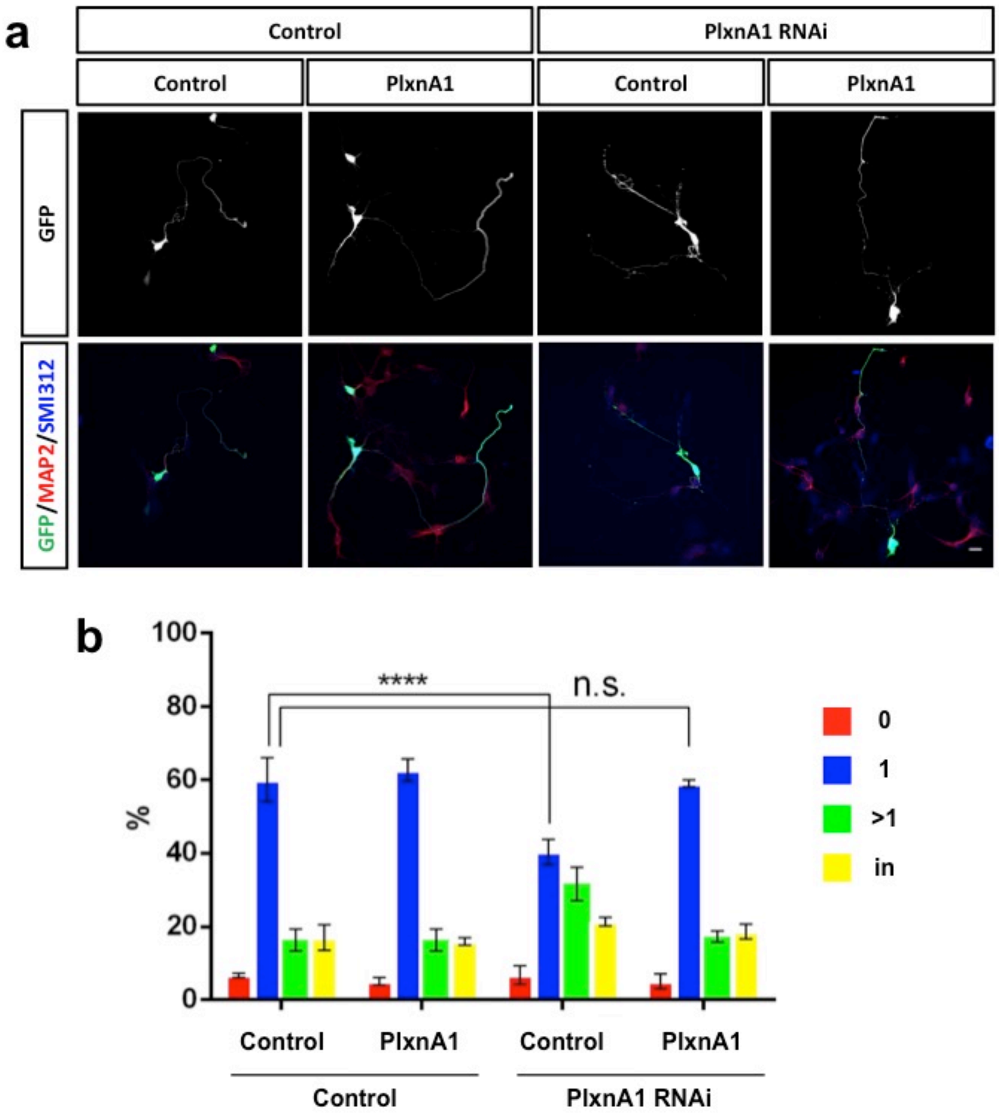

Figure S2. Specificity of the Plexin-A1 knockdown. (**a**) Hippocampal neurons from E18 rat embryos were transfected with vectors for GFP (green), an shRNA against rat Plexin-A1 (PlxnA1 RNAi) or pSUPER (control), and a vector for murine Plexin-A1 (PlxnA1) or pBK-CMV (control) as indicated. The shRNA target site in murine Plexin-A1 contains multiple mismatches in comparison to the rat sequence. Neurons were analyzed at 3 d.i.v. by staining with an anti-MAP2 (red, dendrites) and the SMI-312 antibody (blue, axon). Representative images of transfected neurons are shown. The scale bar is 50 μm. (**b**) The percentage of unpolarized neurons without an axon (0, red), polarized neurons with a single axon (1, blue), neurons with multiple axons (>1, green) and neurons with multiple neurites that are positive for both axonal and dendritic markers (in, yellow) is shown (Tukey's multiple comparisons test and two-way ANOVA; n=3, independent experiments with 100 neurons per experiment; values are means ± s.e.m; p> 0.05, not significant (n.s.), **** p<0.0001 compared to control as indicated).

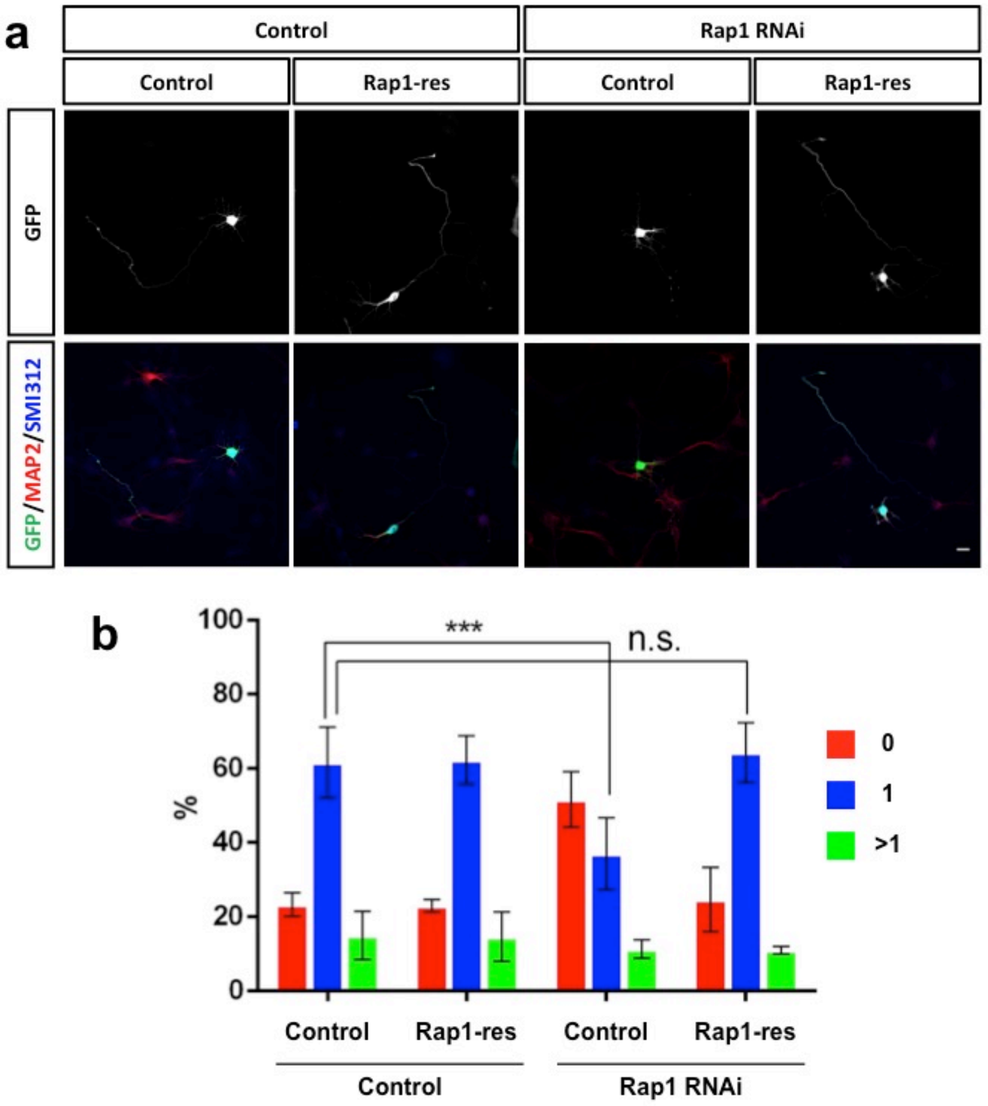

Figure S3. Specificity of the Rap1B knockdown. (**a**) Hippocampal neurons from E18 rat embryos were transfected with vectors for GFP (green), an shRNA against Rap1B (Rap1 RNAi) or pSHAG-1 (control), and a vector for RNAi-resistant Rap1B (Rap1B-res) or pBK-CMV (control) as indicated. Neurons were analyzed at 3 d.i.v. by staining with an anti-MAP2 (red, dendrites) and the SMI-312 antibody (blue, axon). Representative images of transfected neurons are shown. The scale bar is 50 µm. (**b**) The percentage of unpolarized neurons without an axon (0, red), polarized neurons with a single axon (1, blue) and neurons with multiple axons (>1, green) is shown (Tukey's multiple comparisons test and two-way ANOVA; n=3, independent experiments with 100 neurons per experiment; values are means ± s.e.m; $p > 0.05$, not significant (n.s.), *** $p < 0.001$ compared to control as indicated).

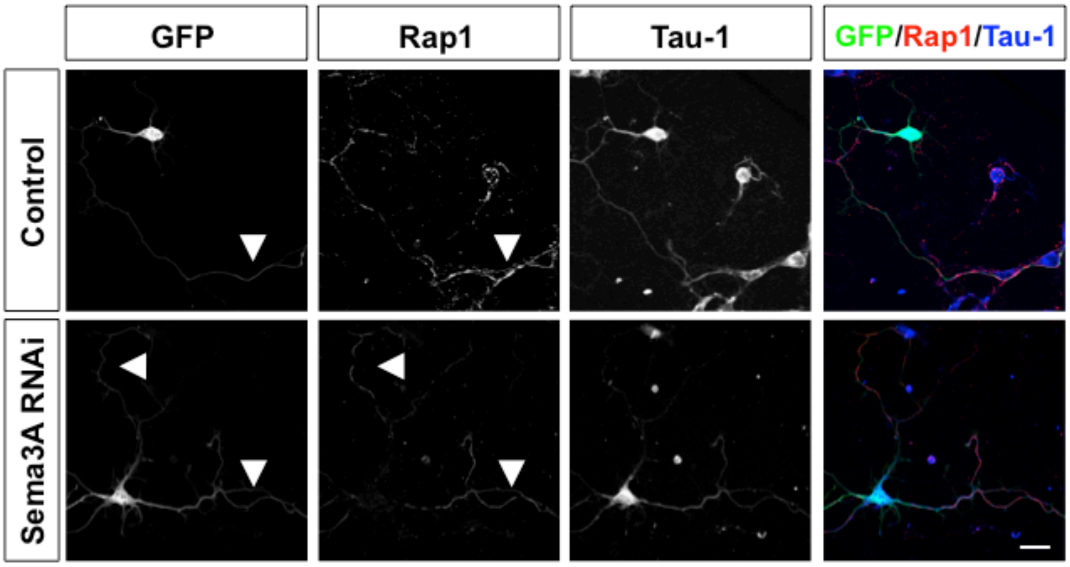

Figure S4. Supernumerary axons formed after knockdown of Sema3A are positive for Rap1. Hippocampal neurons from E18 rat embryos were transfected with vectors for GFP (green) and an shRNA against Sema3A (Sema3A RNAi) or pcDNA6.2-GW/EmGFP-miR (control) and stained at 3 d.i.v. with an anti-Rap1 (red) and the Tau-1 antibody (blue). The arrowhead marks Rap1-positve axons.

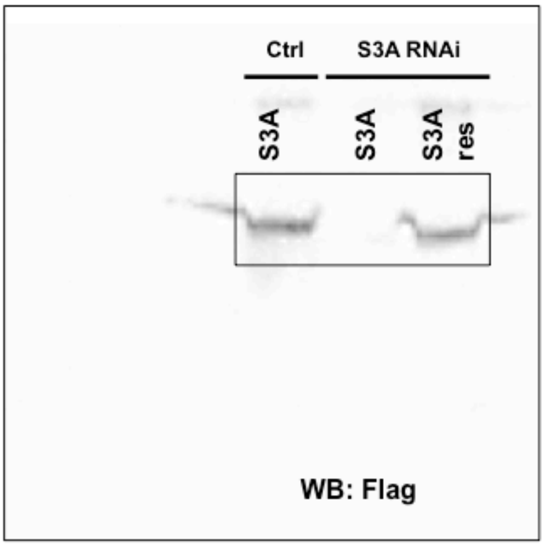

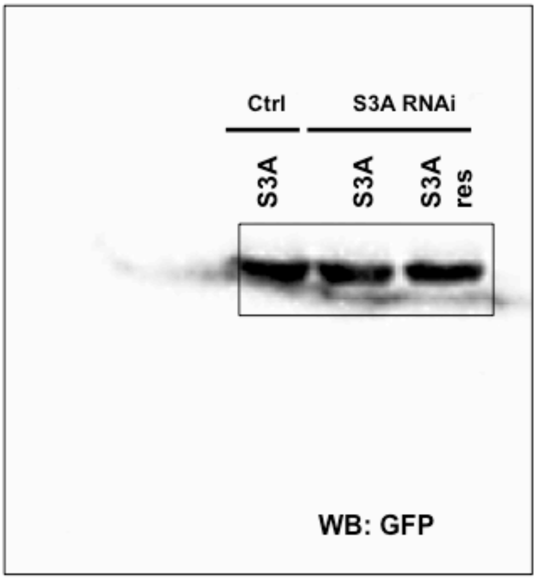

Figure S5. Full-length blots for Figure 2a.
